# Bis(μ-trimethyl­silanolato-κ^2^ *O*:*O*)bis­{[2-(2*H*-benzotriazol-2-yl)-4,6-di-*tert*-pentyl­phenolato-κ^2^ *N*,*O*]zinc}

**DOI:** 10.1107/S1600536811054134

**Published:** 2011-12-23

**Authors:** Hwi Hyun Lee, Min Jeong Go, Ka Hyun Park, Youngjo Kim, Junseong Lee

**Affiliations:** a Department of Chemistry, Chonnam National University, Gwangju 500-757, Republic of Korea; b Department of Chemistry, Chungbuk National University, Cheongju, Chungbuk 361-763, Republic of Korea

## Abstract

The binuclear title complex, [Zn_2_(C_22_H_28_N_3_O)_2_(C_3_H_9_OSi)_2_], has a crystallographic imposed centre of symmetry. The Zn^II^ atom is coordinated by three O and one N atom from one 2-(2*H*-benzotriazol-2-yl)-4,6-di-*tert*-pentyl­phenolate ligand and two bridging trimethyl­silanolate anions in a distorted tetra­hedral geometry. The dihedral angle between the benzotriazole ring system and the benzene ring is 19.83 (5)°. The *tert*-pentyl groups are disordered over two orientations with refined site-occupancy ratios of 0.858 (4):0.142 (4) and 0.665 (6):0.335 (6).

## Related literature

For the use of metal complexes for ring-opening polymerization of cyclic esters, see: Cheng *et al.* (1999 ▶); Chamberlain *et al.* (2001 ▶); Chisholm *et al.* (2001 ▶); Drouin *et al.* (2010 ▶). For metal complexes with bidentate benzotriazol-phenolate ligands, see: Lee *et al.* (2010 ▶, 2011 ▶); Li *et al.* (2011 ▶); Tai *et al.* (2011 ▶).
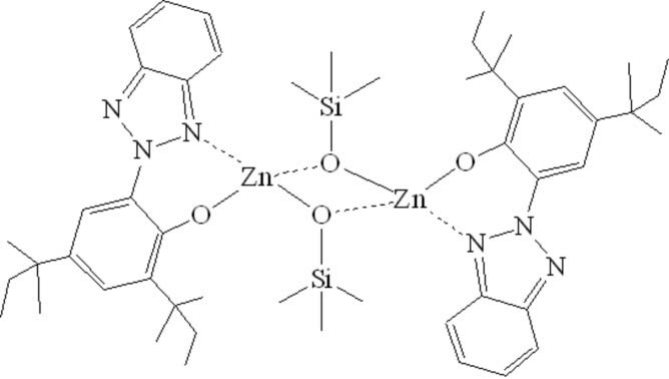


## Experimental

### 

#### Crystal data

[Zn_2_(C_22_H_28_N_3_O)_2_(C_3_H_9_OSi)_2_]
*M* *_r_* = 1010.11
Monoclinic, 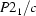

*a* = 10.7640 (4) Å
*b* = 10.7280 (4) Å
*c* = 23.2314 (9) Å
β = 90.597 (2)°
*V* = 2682.53 (18) Å^3^
*Z* = 2
Mo *K*α radiation
μ = 0.99 mm^−1^
*T* = 296 K
0.12 × 0.10 × 0.08 mm

#### Data collection

Bruker SMART 1K CCD diffractometer
Absorption correction: multi-scan (*SADABS*; Bruker, 2004 ▶) *T*_min_ = 0.92, *T*_max_ = 0.95
39361 measured reflections
8000 independent reflections
5487 reflections with *I* > 2σ(*I*)
*R*_int_ = 0.053

#### Refinement

*R*[*F* ^2^ > 2σ(*F* ^2^)] = 0.039
*wR*(*F* ^2^) = 0.108
*S* = 1.01
8000 reflections
387 parameters
18 restraints
H-atom parameters constrained
Δρ_max_ = 0.51 e Å^−3^
Δρ_min_ = −0.40 e Å^−3^

### 

Data collection: *SMART* (Bruker, 2004 ▶); cell refinement: *SAINT* (Bruker, 2004 ▶); data reduction: *SAINT*; program(s) used to solve structure: *SHELXS97* (Sheldrick, 2008 ▶); program(s) used to refine structure: *SHELXL97* (Sheldrick, 2008 ▶); molecular graphics: *ORTEP-3 for Windows* (Farrugia, 1997 ▶); software used to prepare material for publication: *SHELXTL* (Sheldrick, 2008 ▶).

## Supplementary Material

Crystal structure: contains datablock(s) I, global. DOI: 10.1107/S1600536811054134/rz2685sup1.cif

Structure factors: contains datablock(s) I. DOI: 10.1107/S1600536811054134/rz2685Isup2.hkl

Additional supplementary materials:  crystallographic information; 3D view; checkCIF report

## Figures and Tables

**Fig. 1.**
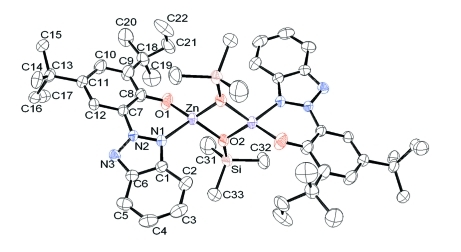
The molecular structure of the title compound with displacement ellipsoids are drawn at the 50% probability level. Unlabelled atoms are related to labelled atoms by the symmetry operation -x, 1 - y, -z. H atoms are omitted for clarity.

## supplementary crystallographic information

### Comment

Due to the environmental concern, the biodegradable poly(cyclic esters) have received increasing attention. The ring opening polymerization (ROP) process provides a convenient and controllable method for the polymerization of cyclic esters. In particular, dinuclear zinc complexes containing two O bridging atoms have attracted considerable attention in the field of organometallic ROP catalysis (Cheng *et al.*, 1999; Drouin *et al.*, 2010; Chisholm *et al.*, 2001; Chamberlain *et al.*, 2001; Li *et al.*, 2011; Tai *et al.*, 2011). We recently reported the synthesis and structural studies of Al and Zr complexes bearing N,*O*-bidentate benzotriazol-phenolate ligands (Lee *et al.*, 2010; Lee *et al.*, 2011). We report herein the synthesis and crystal structure of a new dinuclear zinc(II) silyloxide complex (I) bearing benzotriazolyl ligands, which is a potential catalyst for ring opening polymerization of cyclic esters.

In (I) (Fig. 1), the complex molecule has crystallographically imposed centre of symmetry. Each zinc atom is coordinated by the oxygen atoms of two bridging trimethylsilyloxide anions and by a N,*O*-bidentate benzotriazol-phenolate ligand, resulting in a distorted tetrahedral geometry. The distances Zn—N (2.0077 (14) Å) and Zn—O (1.8822 (16)–1.9609 (15) Å) are a little shorter than those reported for related compounds (Li *et al.*, 2011; Tai *et al.*, 2011). Moreover, a relatively short Zn···Zn contact is observed (2.8402 (4) Å). The Zn—O—Zn angle is significantly narrow (93.24 (6) °) and slightly smaller than that observed for a previously reported compound (Cheng *et al.*, 1999). The benzotrazole ring system and the benzene ring are almost coplanar, forming a dihedral angle of 19.83 (5)°. The crystal packing is governed only by van der Waals interactions.

### Experimental

The title compound was synthesized by the reaction of bis(trimethylsilyl) oxide (0.21 ml, 1 mmol) and [2-(2*H*-benzotriazol-2-yl)–4,6-di-*tert*-pentylphenolato]zinc di(trimethylsilyl)amide (0.58 g, 1 mmol) in dichloromethane solution (30 ml). Crystals suitable for X-ray analysis were obtained by slow evaporation of the resulting solution in refrigerator.

### Refinement

The disordered two *t*-pentyl groups were modeled by splitting the atoms into two sets of two components [C14—C17 and C14A—C17A; C18—C22 and C18A—C22A], the refined site occupation ratios of which were 0.858 (4):0.142 (4) and 0.665 (6):0.335 (6), respectively. H atoms were positioned geometrically and refined using a riding model, with C—H distances fixed to 0.96 (methyl CH_3_), 0.97 (methylene CH_2_) and with *U*_iso_(H) = 1.2*U*_eq_(C) or 1.5*U*_eq_(C) for methyl groups. Bond lengths involving the C14A–C22A atoms were restained to be equal to those of the C14–C22 atoms (SAME instruction in *SHELXL97*). Atom C17A was restrained to be approximately isotropic with a effective standard deviation of 0.01.

### Crystal data

**Table:**

[Zn_2_(C_22_H_28_N_3_O)_2_(C_3_H_9_OSi)_2_]	*F*(000) = 1072
*M**_r_* = 1010.11	*D*_x_ = 1.251 Mg m^−^^3^
Monoclinic, *P*2_1_/*c*	Mo *K*α radiation, λ = 0.71073 Å
Hall symbol: -P 2ybc	Cell parameters from 4477 reflections
*a* = 10.7640 (4) Å	θ = 1.9–30.9°
*b* = 10.7280 (4) Å	µ = 0.99 mm^−^^1^
*c* = 23.2314 (9) Å	*T* = 296 K
β = 90.597 (2)°	Block, yellow
*V* = 2682.53 (18) Å^3^	0.12 × 0.10 × 0.08 mm
*Z* = 2	

### Data collection

**Table:**

Bruker SMART 1K CCD diffractometer	8000 independent reflections
Radiation source: fine-focus sealed tube	5487 reflections with *I* > 2σ(*I*)
graphite	*R*_int_ = 0.053
profile data from /ω scans	θ_max_ = 30.4°, θ_min_ = 1.9°
Absorption correction: multi-scan (*SADABS*; Bruker, 2004)	*h* = −15→15
*T*_min_ = 0.92, *T*_max_ = 0.95	*k* = −15→8
39361 measured reflections	*l* = −32→32

### Refinement

**Table:**

Refinement on *F*^2^	Primary atom site location: structure-invariant direct methods
Least-squares matrix: full	Secondary atom site location: difference Fourier map
*R*[*F*^2^ > 2σ(*F*^2^)] = 0.039	Hydrogen site location: inferred from neighbouring sites
*wR*(*F*^2^) = 0.108	H-atom parameters constrained
*S* = 1.01	*w* = 1/[σ^2^(*F*_o_^2^) + (0.0487*P*)^2^ + 0.8229*P*] where *P* = (*F*_o_^2^ + 2*F*_c_^2^)/3
8000 reflections	(Δ/σ)_max_ = 0.002
387 parameters	Δρ_max_ = 0.51 e Å^−^^3^
18 restraints	Δρ_min_ = −0.40 e Å^−^^3^

### Special details

**Table:**

Geometry. All e.s.d.'s (except the e.s.d. in the dihedral angle between two l.s. planes) are estimated using the full covariance matrix. The cell e.s.d.'s are taken into account individually in the estimation of e.s.d.'s in distances, angles and torsion angles; correlations between e.s.d.'s in cell parameters are only used when they are defined by crystal symmetry. An approximate (isotropic) treatment of cell e.s.d.'s is used for estimating e.s.d.'s involving l.s. planes.
Refinement. Refinement of *F*^2^ against ALL reflections. The weighted *R*-factor *wR* and goodness of fit *S* are based on *F*^2^, conventional *R*-factors *R* are based on *F*, with *F* set to zero for negative *F*^2^. The threshold expression of *F*^2^ > σ(*F*^2^) is used only for calculating *R*-factors(gt) *etc*. and is not relevant to the choice of reflections for refinement. *R*-factors based on *F*^2^ are statistically about twice as large as those based on *F*, and *R*- factors based on ALL data will be even larger.

### Fractional atomic coordinates and isotropic or equivalent isotropic displacement parameters (Å^2^)

**Table:**

	*x*	*y*	*z*	*U*_iso_*/*U*_eq_	Occ. (<1)
Zn	0.104366 (19)	0.55812 (2)	0.026548 (11)	0.04165 (8)	
N1	0.26766 (13)	0.59563 (14)	−0.01068 (7)	0.0341 (3)	
N2	0.35760 (12)	0.66301 (12)	0.01574 (6)	0.0277 (3)	
N3	0.46639 (12)	0.66115 (13)	−0.01036 (6)	0.0311 (3)	
O1	0.15782 (14)	0.62338 (15)	0.09782 (7)	0.0587 (5)	
C1	0.32338 (17)	0.54664 (16)	−0.05741 (8)	0.0337 (4)	
C2	0.2760 (2)	0.4676 (2)	−0.10062 (10)	0.0502 (5)	
H2	0.1938	0.4410	−0.1009	0.060*	
C3	0.3576 (2)	0.4320 (2)	−0.14212 (10)	0.0527 (5)	
H3	0.3300	0.3801	−0.1716	0.063*	
C4	0.4823 (2)	0.4717 (2)	−0.14158 (9)	0.0490 (5)	
H4	0.5346	0.4437	−0.1705	0.059*	
C5	0.52970 (19)	0.54967 (18)	−0.10038 (8)	0.0418 (4)	
H5	0.6120	0.5760	−0.1008	0.050*	
C6	0.44666 (16)	0.58816 (15)	−0.05707 (7)	0.0310 (4)	
C7	0.33891 (15)	0.73715 (15)	0.06658 (7)	0.0295 (3)	
C8	0.24052 (18)	0.71386 (19)	0.10475 (8)	0.0413 (4)	
C9	0.2352 (2)	0.7919 (2)	0.15453 (10)	0.0556 (6)	
C10	0.3235 (2)	0.8842 (2)	0.16192 (9)	0.0473 (5)	
H10	0.3174	0.9354	0.1941	0.057*	
C11	0.42115 (16)	0.90547 (17)	0.12431 (8)	0.0337 (4)	
C12	0.42760 (15)	0.83001 (15)	0.07660 (7)	0.0300 (3)	
H12	0.4919	0.8409	0.0506	0.036*	
C13	0.52264 (18)	1.00306 (17)	0.13694 (9)	0.0385 (4)	
C14	0.5631 (3)	1.0659 (3)	0.07985 (13)	0.0469 (7)	0.858 (4)
H14A	0.6240	1.1290	0.0880	0.070*	0.858 (4)
H14B	0.4921	1.1032	0.0614	0.070*	0.858 (4)
H14C	0.5982	1.0042	0.0548	0.070*	0.858 (4)
C15	0.4803 (2)	1.1039 (2)	0.17774 (13)	0.0487 (7)	0.858 (4)
H15A	0.5445	1.1654	0.1819	0.073*	0.858 (4)
H15B	0.4631	1.0678	0.2146	0.073*	0.858 (4)
H15C	0.4064	1.1424	0.1626	0.073*	0.858 (4)
C16	0.6396 (2)	0.9343 (2)	0.16123 (10)	0.0411 (6)	0.858 (4)
H16A	0.6656	0.8734	0.1330	0.049*	0.858 (4)
H16B	0.7062	0.9947	0.1653	0.049*	0.858 (4)
C17	0.6253 (4)	0.8689 (4)	0.2178 (2)	0.0504 (9)	0.858 (4)
H17A	0.6049	0.9286	0.2470	0.076*	0.858 (4)
H17B	0.7017	0.8280	0.2279	0.076*	0.858 (4)
H17C	0.5600	0.8082	0.2147	0.076*	0.858 (4)
C18	0.1446 (3)	0.7613 (4)	0.20390 (14)	0.0438 (9)	0.665 (6)
C19	0.1599 (8)	0.6321 (8)	0.2290 (3)	0.074 (3)	0.665 (6)
H19A	0.1516	0.5712	0.1989	0.111*	0.665 (6)
H19B	0.0971	0.6182	0.2574	0.111*	0.665 (6)
H19C	0.2405	0.6248	0.2467	0.111*	0.665 (6)
C20	0.1569 (5)	0.8554 (6)	0.2538 (2)	0.0737 (16)	0.665 (6)
H20A	0.0870	0.8471	0.2788	0.111*	0.665 (6)
H20B	0.1593	0.9385	0.2385	0.111*	0.665 (6)
H20C	0.2322	0.8391	0.2751	0.111*	0.665 (6)
C21	0.0133 (4)	0.7707 (5)	0.17880 (18)	0.0579 (12)	0.665 (6)
H21A	−0.0012	0.6995	0.1539	0.069*	0.665 (6)
H21B	−0.0455	0.7657	0.2101	0.069*	0.665 (6)
C22	−0.0119 (8)	0.8872 (9)	0.1452 (4)	0.077 (3)	0.665 (6)
H22A	0.0089	0.9585	0.1684	0.115*	0.665 (6)
H22B	−0.0983	0.8905	0.1347	0.115*	0.665 (6)
H22C	0.0375	0.8875	0.1111	0.115*	0.665 (6)
C14A	0.4359 (15)	1.1429 (13)	0.1393 (7)	0.050 (4)	0.142 (4)
H14D	0.4903	1.2113	0.1483	0.075*	0.142 (4)
H14E	0.3735	1.1364	0.1684	0.075*	0.142 (4)
H14F	0.3967	1.1570	0.1026	0.075*	0.142 (4)
C15A	0.608 (2)	1.017 (2)	0.1012 (8)	0.072 (7)	0.142 (4)
H15D	0.6622	1.0830	0.1137	0.108*	0.142 (4)
H15E	0.5732	1.0371	0.0642	0.108*	0.142 (4)
H15F	0.6549	0.9408	0.0984	0.108*	0.142 (4)
C16A	0.5538 (15)	0.9979 (14)	0.2060 (7)	0.053 (5)	0.142 (4)
H16C	0.6240	1.0516	0.2148	0.064*	0.142 (4)
H16D	0.4829	1.0275	0.2275	0.064*	0.142 (4)
C17A	0.584 (2)	0.865 (2)	0.2231 (15)	0.057 (8)	0.142 (4)
H17D	0.6493	0.8342	0.1994	0.086*	0.142 (4)
H17E	0.5112	0.8144	0.2181	0.086*	0.142 (4)
H17F	0.6099	0.8634	0.2627	0.086*	0.142 (4)
C18A	0.0932 (7)	0.8057 (6)	0.1834 (3)	0.0426 (18)	0.335 (6)
C19A	−0.0218 (17)	0.8360 (16)	0.1471 (10)	0.079 (5)	0.335 (6)
H19D	−0.0211	0.7871	0.1124	0.119*	0.335 (6)
H19E	−0.0216	0.9229	0.1373	0.119*	0.335 (6)
H19F	−0.0951	0.8169	0.1685	0.119*	0.335 (6)
C20A	0.1012 (10)	0.9032 (11)	0.2329 (5)	0.076 (3)	0.335 (6)
H20D	0.0247	0.9034	0.2538	0.115*	0.335 (6)
H20E	0.1153	0.9845	0.2169	0.115*	0.335 (6)
H20F	0.1685	0.8820	0.2585	0.115*	0.335 (6)
C21A	0.0677 (6)	0.6779 (7)	0.2073 (3)	0.053 (2)	0.335 (6)
H21C	−0.0016	0.6830	0.2336	0.063*	0.335 (6)
H21D	0.0436	0.6229	0.1760	0.063*	0.335 (6)
C22A	0.1766 (17)	0.623 (2)	0.2383 (10)	0.104 (8)	0.335 (6)
H22D	0.2393	0.6011	0.2110	0.156*	0.335 (6)
H22E	0.1510	0.5499	0.2588	0.156*	0.335 (6)
H22F	0.2097	0.6830	0.2651	0.156*	0.335 (6)
O2	0.04159 (12)	0.38894 (12)	0.01836 (7)	0.0469 (4)	
Si	0.07437 (5)	0.25351 (5)	0.04705 (3)	0.04524 (15)	
C31	0.1120 (3)	0.2751 (3)	0.12478 (12)	0.0821 (9)	
H31A	0.1790	0.3336	0.1289	0.123*	
H31B	0.1363	0.1967	0.1413	0.123*	
H31C	0.0402	0.3063	0.1443	0.123*	
C32	−0.0656 (2)	0.1519 (2)	0.03794 (14)	0.0664 (7)	
H32A	−0.1308	0.1814	0.0623	0.100*	
H32B	−0.0447	0.0678	0.0483	0.100*	
H32C	−0.0932	0.1542	−0.0015	0.100*	
C33	0.2097 (2)	0.1835 (2)	0.01087 (12)	0.0587 (6)	
H33A	0.1893	0.1679	−0.0288	0.088*	
H33B	0.2312	0.1063	0.0294	0.088*	
H33C	0.2789	0.2397	0.0132	0.088*	

### Atomic displacement parameters (Å^2^)

**Table:**

	*U*^11^	*U*^22^	*U*^33^	*U*^12^	*U*^13^	*U*^23^
Zn	0.02557 (11)	0.03335 (12)	0.06619 (17)	−0.00948 (8)	0.00779 (9)	−0.01189 (10)
N1	0.0250 (7)	0.0330 (7)	0.0445 (9)	−0.0044 (6)	0.0011 (6)	−0.0085 (6)
N2	0.0242 (6)	0.0257 (7)	0.0334 (7)	−0.0041 (5)	0.0036 (5)	−0.0012 (6)
N3	0.0256 (7)	0.0323 (8)	0.0356 (8)	−0.0021 (5)	0.0073 (6)	0.0020 (6)
O1	0.0534 (9)	0.0619 (10)	0.0614 (10)	−0.0350 (8)	0.0272 (7)	−0.0197 (8)
C1	0.0347 (9)	0.0295 (9)	0.0368 (9)	0.0014 (7)	0.0009 (7)	−0.0044 (7)
C2	0.0486 (12)	0.0468 (12)	0.0550 (13)	−0.0027 (9)	−0.0036 (10)	−0.0172 (10)
C3	0.0704 (15)	0.0440 (12)	0.0437 (12)	0.0053 (10)	−0.0009 (10)	−0.0134 (9)
C4	0.0694 (15)	0.0422 (11)	0.0358 (11)	0.0127 (10)	0.0137 (10)	0.0002 (9)
C5	0.0446 (11)	0.0417 (10)	0.0394 (10)	0.0054 (8)	0.0142 (8)	0.0062 (9)
C6	0.0343 (9)	0.0271 (8)	0.0318 (9)	0.0015 (6)	0.0058 (7)	0.0033 (7)
C7	0.0276 (8)	0.0294 (8)	0.0316 (9)	−0.0042 (6)	0.0037 (6)	−0.0028 (7)
C8	0.0391 (10)	0.0416 (10)	0.0436 (11)	−0.0146 (8)	0.0133 (8)	−0.0064 (8)
C9	0.0557 (13)	0.0615 (14)	0.0503 (12)	−0.0258 (11)	0.0254 (10)	−0.0175 (11)
C10	0.0538 (12)	0.0485 (12)	0.0398 (11)	−0.0158 (9)	0.0125 (9)	−0.0150 (9)
C11	0.0323 (9)	0.0307 (9)	0.0381 (10)	−0.0039 (7)	0.0002 (7)	−0.0020 (7)
C12	0.0249 (8)	0.0293 (8)	0.0358 (9)	−0.0043 (6)	0.0033 (6)	0.0014 (7)
C13	0.0378 (10)	0.0320 (9)	0.0454 (11)	−0.0069 (7)	−0.0053 (8)	−0.0043 (8)
C14	0.0487 (15)	0.0391 (15)	0.0529 (18)	−0.0178 (11)	−0.0057 (12)	0.0055 (12)
C15	0.0412 (13)	0.0354 (12)	0.0696 (19)	−0.0012 (10)	−0.0023 (12)	−0.0172 (12)
C16	0.0363 (11)	0.0396 (12)	0.0472 (14)	0.0009 (9)	−0.0062 (9)	−0.0127 (10)
C17	0.052 (2)	0.0496 (17)	0.0491 (19)	0.0103 (15)	−0.0082 (18)	−0.0078 (13)
C18	0.0370 (19)	0.059 (2)	0.0358 (18)	−0.0071 (15)	0.0102 (14)	0.0003 (15)
C19	0.089 (7)	0.079 (4)	0.054 (3)	−0.005 (4)	0.023 (3)	0.021 (3)
C20	0.068 (3)	0.110 (4)	0.044 (2)	−0.024 (3)	0.029 (2)	−0.023 (3)
C21	0.031 (2)	0.086 (3)	0.057 (2)	−0.0003 (19)	0.0150 (16)	−0.009 (2)
C22	0.041 (3)	0.123 (7)	0.067 (4)	0.032 (4)	0.007 (2)	0.011 (4)
C14A	0.061 (10)	0.029 (7)	0.060 (10)	−0.009 (6)	−0.018 (8)	0.009 (7)
C15A	0.093 (17)	0.076 (15)	0.046 (12)	−0.060 (13)	0.000 (10)	−0.024 (10)
C16A	0.046 (9)	0.050 (9)	0.064 (11)	0.008 (7)	−0.014 (7)	−0.016 (8)
C17A	0.054 (11)	0.062 (10)	0.055 (10)	0.019 (8)	−0.010 (8)	0.004 (7)
C18A	0.027 (4)	0.060 (4)	0.041 (4)	0.003 (3)	0.011 (3)	−0.004 (3)
C19A	0.048 (6)	0.094 (12)	0.097 (10)	0.004 (7)	0.024 (6)	0.021 (9)
C20A	0.064 (6)	0.108 (8)	0.058 (6)	−0.001 (5)	0.033 (5)	−0.025 (6)
C21A	0.040 (4)	0.075 (5)	0.044 (4)	0.008 (3)	0.011 (3)	0.014 (3)
C22A	0.036 (6)	0.131 (12)	0.146 (15)	0.010 (6)	−0.017 (7)	0.102 (11)
O2	0.0287 (7)	0.0296 (7)	0.0825 (11)	−0.0064 (5)	0.0036 (6)	−0.0067 (7)
Si	0.0301 (3)	0.0374 (3)	0.0683 (4)	−0.0064 (2)	0.0056 (2)	−0.0033 (3)
C31	0.0734 (19)	0.103 (2)	0.0703 (18)	−0.0146 (17)	0.0130 (15)	−0.0026 (17)
C32	0.0414 (12)	0.0404 (12)	0.117 (2)	−0.0124 (9)	−0.0017 (13)	0.0114 (13)
C33	0.0401 (12)	0.0518 (13)	0.0841 (17)	0.0052 (9)	0.0028 (11)	−0.0113 (12)

### Geometric parameters (Å, °)

**Table:**

Zn—O1	1.8822 (16)	C19—H19A	0.9600
Zn—O2	1.9453 (13)	C19—H19B	0.9600
Zn—O2^i^	1.9609 (15)	C19—H19C	0.9600
Zn—N1	2.0077 (14)	C20—H20A	0.9600
Zn—Zn^i^	2.8402 (4)	C20—H20B	0.9600
N1—N2	1.3507 (19)	C20—H20C	0.9600
N1—C1	1.352 (2)	C21—C22	1.497 (9)
N2—N3	1.3245 (18)	C21—H21A	0.9700
N2—C7	1.440 (2)	C21—H21B	0.9700
N3—C6	1.353 (2)	C22—H22A	0.9600
O1—C8	1.326 (2)	C22—H22B	0.9600
C1—C6	1.400 (2)	C22—H22C	0.9600
C1—C2	1.406 (3)	C14A—H14D	0.9600
C2—C3	1.365 (3)	C14A—H14E	0.9600
C2—H2	0.9300	C14A—H14F	0.9600
C3—C4	1.408 (3)	C15A—H15D	0.9600
C3—H3	0.9300	C15A—H15E	0.9600
C4—C5	1.366 (3)	C15A—H15F	0.9600
C4—H4	0.9300	C16A—C17A	1.510 (18)
C5—C6	1.414 (2)	C16A—H16C	0.9700
C5—H5	0.9300	C16A—H16D	0.9700
C7—C12	1.398 (2)	C17A—H17D	0.9600
C7—C8	1.411 (2)	C17A—H17E	0.9600
C8—C9	1.429 (3)	C17A—H17F	0.9600
C9—C10	1.383 (3)	C18A—C21A	1.506 (9)
C9—C18	1.548 (3)	C18A—C19A	1.526 (16)
C9—C18A	1.682 (7)	C18A—C20A	1.556 (10)
C10—C11	1.392 (3)	C19A—H19D	0.9600
C10—H10	0.9300	C19A—H19E	0.9600
C11—C12	1.375 (2)	C19A—H19F	0.9600
C11—C13	1.539 (2)	C20A—H20D	0.9600
C12—H12	0.9300	C20A—H20E	0.9600
C13—C15A	1.255 (18)	C20A—H20F	0.9600
C13—C15	1.512 (3)	C21A—C22A	1.490 (13)
C13—C14	1.554 (4)	C21A—H21C	0.9700
C13—C16	1.560 (3)	C21A—H21D	0.9700
C13—C16A	1.636 (15)	C22A—H22D	0.9600
C13—C14A	1.768 (15)	C22A—H22E	0.9600
C14—H14A	0.9600	C22A—H22F	0.9600
C14—H14B	0.9600	O2—Si	1.6354 (15)
C14—H14C	0.9600	O2—Zn^i^	1.9609 (15)
C15—H15A	0.9600	Si—C33	1.849 (2)
C15—H15B	0.9600	Si—C31	1.861 (3)
C15—H15C	0.9600	Si—C32	1.870 (2)
C16—C17	1.498 (5)	C31—H31A	0.9600
C16—H16A	0.9700	C31—H31B	0.9600
C16—H16B	0.9700	C31—H31C	0.9600
C17—H17A	0.9600	C32—H32A	0.9600
C17—H17B	0.9600	C32—H32B	0.9600
C17—H17C	0.9600	C32—H32C	0.9600
C18—C19	1.512 (9)	C33—H33A	0.9600
C18—C21	1.527 (5)	C33—H33B	0.9600
C18—C20	1.542 (5)	C33—H33C	0.9600
			
O1—Zn—O2	122.40 (7)	C22—C21—C18	114.6 (5)
O1—Zn—O2^i^	126.59 (7)	C22—C21—H21A	108.6
O2—Zn—O2^i^	86.71 (6)	C18—C21—H21A	108.6
O1—Zn—N1	92.49 (6)	C22—C21—H21B	108.6
O2—Zn—N1	116.72 (6)	C18—C21—H21B	108.6
O2^i^—Zn—N1	114.39 (6)	H21A—C21—H21B	107.6
O1—Zn—Zn^i^	141.13 (4)	C13—C14A—H14D	109.5
O2—Zn—Zn^i^	43.57 (4)	C13—C14A—H14E	109.5
O2^i^—Zn—Zn^i^	43.14 (4)	H14D—C14A—H14E	109.5
N1—Zn—Zn^i^	126.37 (5)	C13—C14A—H14F	109.5
N2—N1—C1	104.54 (14)	H14D—C14A—H14F	109.5
N2—N1—Zn	122.55 (11)	H14E—C14A—H14F	109.5
C1—N1—Zn	131.73 (12)	C13—C15A—H15D	109.5
N3—N2—N1	114.63 (13)	C13—C15A—H15E	109.5
N3—N2—C7	121.05 (13)	H15D—C15A—H15E	109.5
N1—N2—C7	124.25 (13)	C13—C15A—H15F	109.5
N2—N3—C6	104.11 (13)	H15D—C15A—H15F	109.5
C8—O1—Zn	125.31 (13)	H15E—C15A—H15F	109.5
N1—C1—C6	107.47 (15)	C17A—C16A—C13	109.3 (17)
N1—C1—C2	130.33 (18)	C17A—C16A—H16C	109.8
C6—C1—C2	122.20 (18)	C13—C16A—H16C	109.8
C3—C2—C1	116.2 (2)	C17A—C16A—H16D	109.8
C3—C2—H2	121.9	C13—C16A—H16D	109.8
C1—C2—H2	121.9	H16C—C16A—H16D	108.3
C2—C3—C4	122.0 (2)	C16A—C17A—H17D	109.5
C2—C3—H3	119.0	C16A—C17A—H17E	109.5
C4—C3—H3	119.0	H17D—C17A—H17E	109.5
C5—C4—C3	122.71 (19)	C16A—C17A—H17F	109.5
C5—C4—H4	118.6	H17D—C17A—H17F	109.5
C3—C4—H4	118.6	H17E—C17A—H17F	109.5
C4—C5—C6	116.30 (19)	C21A—C18A—C19A	104.4 (8)
C4—C5—H5	121.8	C21A—C18A—C20A	110.4 (7)
C6—C5—H5	121.8	C19A—C18A—C20A	107.7 (10)
N3—C6—C1	109.24 (15)	C21A—C18A—C9	103.7 (5)
N3—C6—C5	130.11 (17)	C19A—C18A—C9	122.2 (9)
C1—C6—C5	120.61 (17)	C20A—C18A—C9	108.0 (6)
C12—C7—C8	122.51 (16)	C18A—C19A—H19D	109.5
C12—C7—N2	115.43 (14)	C18A—C19A—H19E	109.5
C8—C7—N2	122.01 (15)	H19D—C19A—H19E	109.5
O1—C8—C7	124.11 (17)	C18A—C19A—H19F	109.5
O1—C8—C9	119.63 (16)	H19D—C19A—H19F	109.5
C7—C8—C9	116.22 (16)	H19E—C19A—H19F	109.5
C10—C9—C8	119.11 (17)	C18A—C20A—H20D	109.5
C10—C9—C18	119.8 (2)	C18A—C20A—H20E	109.5
C8—C9—C18	120.4 (2)	H20D—C20A—H20E	109.5
C10—C9—C18A	121.0 (3)	C18A—C20A—H20F	109.5
C8—C9—C18A	114.7 (3)	H20D—C20A—H20F	109.5
C9—C10—C11	124.20 (18)	H20E—C20A—H20F	109.5
C9—C10—H10	117.9	C22A—C21A—C18A	113.1 (11)
C11—C10—H10	117.9	C22A—C21A—H21C	109.0
C12—C11—C10	117.02 (16)	C18A—C21A—H21C	109.0
C12—C11—C13	120.84 (16)	C22A—C21A—H21D	109.0
C10—C11—C13	122.04 (16)	C18A—C21A—H21D	109.0
C11—C12—C7	120.92 (15)	H21C—C21A—H21D	107.8
C11—C12—H12	119.5	C21A—C22A—H22D	109.5
C7—C12—H12	119.5	C21A—C22A—H22E	109.5
C15A—C13—C15	124.0 (9)	H22D—C22A—H22E	109.5
C15A—C13—C11	118.6 (7)	C21A—C22A—H22F	109.5
C15—C13—C11	112.85 (17)	H22D—C22A—H22F	109.5
C15—C13—C14	108.3 (2)	H22E—C22A—H22F	109.5
C11—C13—C14	109.68 (17)	Si—O2—Zn	135.70 (9)
C15—C13—C16	111.04 (18)	Si—O2—Zn^i^	129.97 (8)
C11—C13—C16	108.42 (16)	Zn—O2—Zn^i^	93.29 (6)
C14—C13—C16	106.35 (19)	O2—Si—C33	110.09 (10)
C15A—C13—C16A	120.5 (11)	O2—Si—C31	109.18 (13)
C11—C13—C16A	107.6 (6)	C33—Si—C31	109.09 (14)
C14—C13—C16A	142.6 (6)	O2—Si—C32	107.56 (10)
C15A—C13—C14A	108.2 (13)	C33—Si—C32	110.43 (12)
C11—C13—C14A	102.1 (5)	C31—Si—C32	110.46 (14)
C16—C13—C14A	144.5 (5)	Si—C31—H31A	109.5
C13—C14—H14A	109.5	Si—C31—H31B	109.5
C13—C14—H14B	109.5	H31A—C31—H31B	109.5
C13—C14—H14C	109.5	Si—C31—H31C	109.5
C13—C15—H15A	109.5	H31A—C31—H31C	109.5
C13—C15—H15B	109.5	H31B—C31—H31C	109.5
C13—C15—H15C	109.5	Si—C32—H32A	109.5
C17—C16—C13	116.6 (2)	Si—C32—H32B	109.5
C17—C16—H16A	108.1	H32A—C32—H32B	109.5
C13—C16—H16A	108.1	Si—C32—H32C	109.5
C17—C16—H16B	108.1	H32A—C32—H32C	109.5
C13—C16—H16B	108.1	H32B—C32—H32C	109.5
H16A—C16—H16B	107.3	Si—C33—H33A	109.5
C19—C18—C21	107.7 (5)	Si—C33—H33B	109.5
C19—C18—C20	107.6 (4)	H33A—C33—H33B	109.5
C21—C18—C20	108.4 (3)	Si—C33—H33C	109.5
C19—C18—C9	114.5 (4)	H33A—C33—H33C	109.5
C21—C18—C9	106.9 (3)	H33B—C33—H33C	109.5
C20—C18—C9	111.6 (3)		

Symmetry codes: (i) −*x*, −*y*+1, −*z*.
